# The Role of Subjective Age Views for 9-Year Change in Self-Reported Problems With Vision and Hearing

**DOI:** 10.1093/geroni/igaa057.1971

**Published:** 2020-12-16

**Authors:** Markus Wettstein, Hans-Werner Wahl, Svenja Spuling

**Affiliations:** 1 German Centre of Gerontology, Berlin, Germany; 2 University of Heidelberg, Heidelberg, Baden-Wurttemberg, Germany; 3 German Centre of Gerontology, Berlin, Berlin, Germany

## Abstract

We examined the role of subjective age views (subjective age; attitudes toward own aging [ATOA]; aging-related cognitions, comprising continuous growth, social loss, and physical decline) for changes in self-reported problems with vision and hearing over up to 9 years. A subsample of the German Ageing Survey (2,499 adults aged 60-85 years at baseline) was investigated. Controlling for gender, age, education, self-rated health, and region of residence (West vs. East Germany), a younger subjective age at baseline predicted less steep increase in vision problems among individuals who were chronologically older at baseline. More favorable ATOA scores were associated with less increase in hearing problems. Higher scores on continuous growth went along with less increase in hearing problems, whereas higher social loss scores were associated with a steeper increase in vision problems. Several associations increased with advancing age. Our findings suggest that subjective age views indeed predict late-life changes in sensory problems.

